# Analysis of TTSuV1b antibody in porcine serum and its correlation with four antibodies against common viral infectious diseases

**DOI:** 10.1186/s12985-015-0349-6

**Published:** 2015-08-12

**Authors:** Zhongsheng Li, Jingxin Qiao, Yonglong He, Yiwen Chen, Guiping Wang

**Affiliations:** Department of veterinary research, Guangdong Haid Institute of Animal Husbandry and Veterinary, Panyu District, Fuping Road, Guangzhou, 511440 China

## Abstract

**Background:**

The purpose of the present study was to evaluate the correlation between Torque teno sus virus 1b (TTSuV1b) infection and other viral infections or vaccine immunization in conventional pigs.

**Methods:**

With overexpressed and purified viral protein TTSuV1b as antigen, an indirect enzyme-linked immunosorbent assay (ELISA) method for detecting TTSuV1b antibody was established, which demonstrated great specificity and reproducibility. Porcine serum samples (n = 212) were tested using ELISA. Meanwhile, the antibodies against Classical Swine Fever Virus (CSFV), Pseudorabies Virus (PRV), Porcine Reproductive and Respiratory Syndrome Virus (PRRSV), and Porcine Circovirus 2 (PCV2) were also examined using the commercial kits.

**Results:**

Statistical analysis indicated that the level of anti-TTSuV1b antibody was positively correlated with the level of anti-PCV2 antibody in a lesser extent; the level of antibodies against TTSuV1b or PCV2 were significantly lower in porcine serum with low level of TTSuV1b virus, implicating the potential consistency and synchronization in the mechanism of TTSuV1b and PCV2 infection. Whereas, antibodies against PRRSV or CSFV showed no statistical significance on comparison with anti-TTSuV1b antibody, implicating that in conventional pigs, the antibody level for PRRSV and CSFV were not significantly influenced by TTSuV1b infection.

**Conclusion:**

In conclusion, examination of anti-TTSuV1b antibody in porcine serum with the presently established ELISA method would serve as a supplementary approach for etiological investigation, and the combined statistical analysis of the antibodies against four other viruses might help to further understand the TTSuV1b infection as well as its pathogenicity.

## Background

Torque teno virus (TTV) is an icosahedral, circular, negative single-stranded deoxyribonucleic acid (ssDNA) virus without envelope. The diameter of this virus particle is 30–32 nm as observed under an electron microscope. TTV is widely distributed in plenty of domestic animals including pig, cow, goat, dog, cat, and poultry, and in wild animals [1–3]. According to the 9th report of the International Committee on Taxonomy of Viruses (ICTV) in 2009, Torque teno sus virus (TTSuV), the TTV which infects pigs, belongs to the family of Anelloviridae and the genus of Iotatorquevirus, and it is composed of two species: Torque teno sus virus 1 (TTSuV1) and Torque teno sus virus 2 (TTSuV2). In 2011, a new genus Kappatorquevirus, including a species of Torque teno sus virus k2, was supplemented by ICTV; later, the TTSuV1 and TTSuV2 were renamed TTSuV1a and TTSuV1b respectively, in relation to newly added Torque teno sus virus k2 [4].

The full length of TTSuVs is 2900 bp, which composed of 3 open reading frames (ORFs): ORF1, ORF2, and ORF3 (alternatively called as ORF2/2). Through comparison with related ssDNA viruses, it is assumed that the capsid protein of TTV ORF1 with a length of 1875–1884 bp is the sole protein structure [3, 5–8]. TTV ORF2 has a domain similar to protein tyrosine kinase, which might be related to the regulation or replication of proteins in cells or viruses during infection period [9, 10]. The precise function of TTV ORF3 is still elusive.

Abundant epidemiological investigations confirmed that TTSuVs exhibited high infection rate in pigs. McKeown et al. had investigated 154 samples of porcine serum obtained from various countries including China, and found that the positive rate of TTSuVs were 66.2 % (102/154) [11]. Since 2009, TTSuVs have been subjected to molecular epidemiological investigations by plenty of research institutions in China, and it has been found that the TTSuVs infection in pigs is a general phenomenon, with an infection rate between 47.4 and 87.1 % [12–15]. In 2014, Leblanc et al. examined the existence of TTSuVs in commercial pigs in Canada through real-time polymerase chain reaction (PCR), and found that the detectable rate of TTSuVs in pigs was 97.9 %, with a maximum viral load of 9.9 × 105 copies per gram and the detectable rate in liver was 98.6 %, with a maximum viral load of 9.9 × 106 copies per gram [16]. TTSuVs are also found human. Jimenez-Melsio et al. found that 25 % of the human embryo samples were TTSuV-positive [17]. The infection rate of subtypes TTSuV1a and TTSuV1b are almost same in pigs; in some farms, it has been found that TTSuV1a have a higher infection rate than that of TTSuV1b [11, 18]. Despite of the high infection rate in pigs, it seemed that TTSuVs have no direct pathogenicity to the pigs; the incidence of TTSuVs in pigs and their infection mechanism were poorly understood. Statistical analysis indicated that TTSuVs might be associated with Postweaning Multisystemic Wasting Syndrome (PMWS) and Porcine Dermatitis and Nephropathy Syndrome (PDNS) and the synergistic effect between TTSuV infection and other pathogens might lead to a more severe disease [5].

In the present study, the viral DNA from a TTSuV1b strain, whose sequence had already been confirmed, was used as the template to clone TTSuV1b ORF1, which encodes the viral capsid protein. By using recombinant protein as the antigen, TTSuV1b antibody titers were measured and analyzed in 212 samples of porcine serum using an enzyme-linked immunosorbent assay (ELISA). The antibodies levels of Classical Swine Fever Virus (CSFV), Pseudorabies Virus (PRV), Porcine Reproductive and Respiratory Syndrome Virus (PRRSV), or Porcine Circovirus 2 (PCV2) were determined using the commercially available Kits and analyzed the potential correlation between the levels of TTSuV1b antibody and the antibodies against the other four viruses.

## Results

### Construction of the expression plasmid and homology analysis of the recombinant protein

**Fig. 1:**
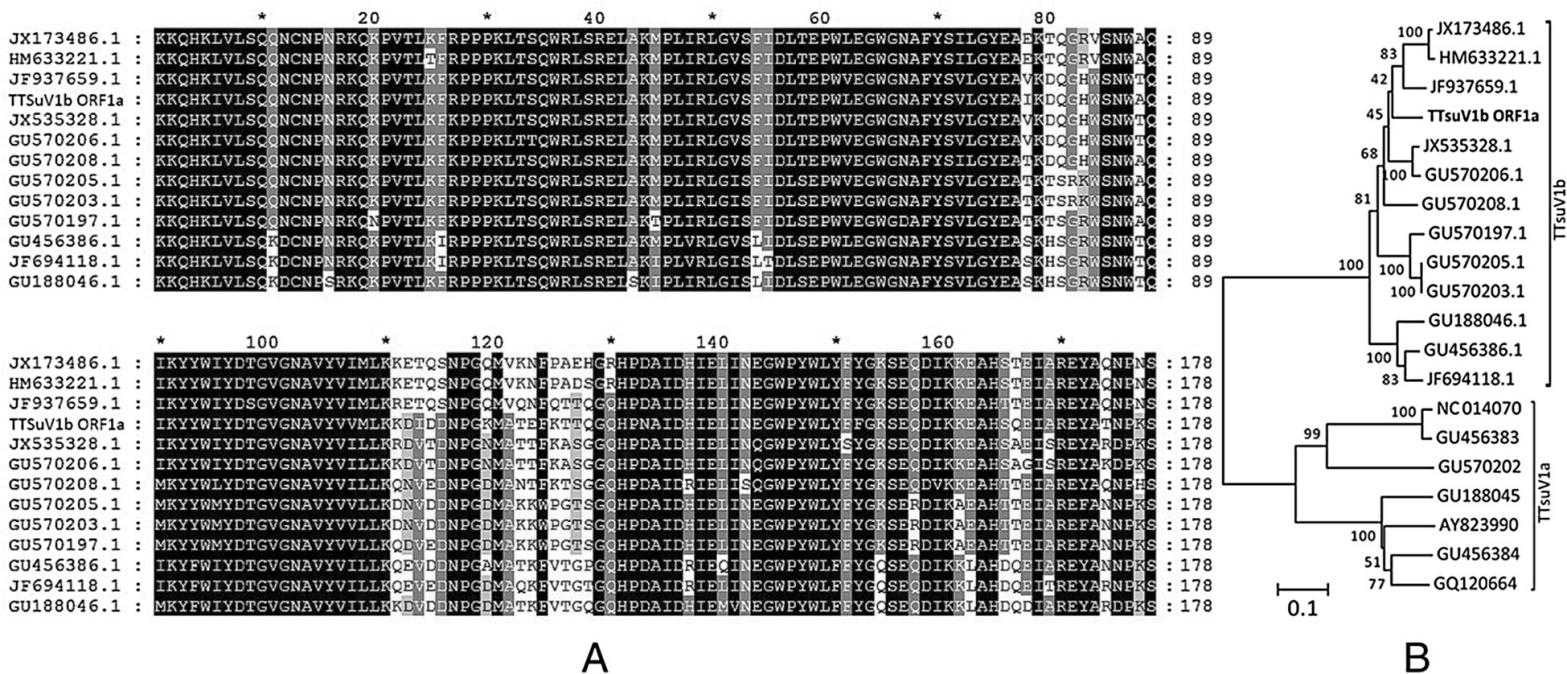
Alignments of amino acid sequence of TTSuV1b ORF1a (**a**) and phylogenetic analysis (**b**). Sequence name are in GeneBank registration number. The neighbor-joining tree was constructed using amino acid sequences through MEGA 6 software package. The bootstrap values are indicated near the branch. The scale bar represents 0.1 amino acid substitutions per site

The recombinant plasmid *TTSuV1b ORF1a-*PET21b was successfully constructed, which was further verified by sequencing. Homology analysis of TTSuV1b ORF1a polypeptide showed high homology to the corresponding area of several TTSuV1b strains (79.7–87.8 %, Fig. 1), but shared seldom homology to the proximate segment of TTSuV1a strains (10.67–17.98 %, Fig. 1-b). The phylogenetic development of viral proteins was analyzed and found that the sequence for TTSuV1b ORF1a, along with those for other strains reported previously in China such as JF937659.1, HM633221.1, and JX173486.1, were in the same branch, implicating a closer evolutionary relationship; meanwhile, the sequence analysis also showed a closer evolutionary relationship with viral strains reported previously in Spain such as GU570208.1, GU570206.1, GU570205.1, and GU570203.1 (Fig. 1-b). The sequence analysis showed 80.9 and 81.46 % homology to that of the strains GU188046.1 (Germany) and GU456386.1 (USA), respectively. Owing to the relative high homology of TTSuV1b ORF1a to the corresponding area of previously reported TTSuV1b strains, this polypeptide should be a good representative for research of TTSuV1b antigenicity.

### Protein expression and purification of the recombinant TTSuV1b ORF1a

**Fig. 2:**
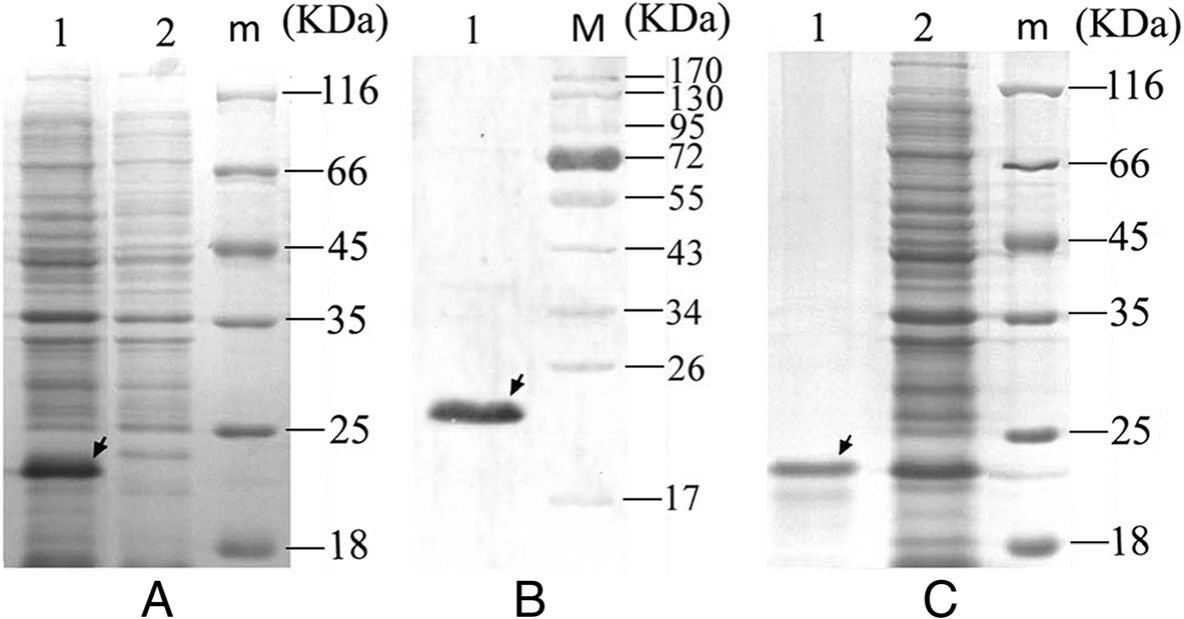
SDS-PAGE (**a**, **c**) and Western blot (**b**) analysis of recombinant TTSuV1b ORF1a. **a** Lane 1–2: SDS-PAGE of Prokaryotic expression production of *TTSuV1b ORF1a*-pET21 and the control vector; m: protein ladder. **b** Lane 1: Western-Blotting of the TSuV2 ORF1a recombinant proteins; M: prestained protein ladder. **c** Lane 1: SDS-PAGE of purified TSuV2 ORF1a recombinant proteins; Lane 2: SDS-PAGE of unpurified TSuV2 ORF1a recombinant proteins; M: protein ladder. The targets are all marked with arrows

The recombinant engineering of bacteria *TTSuV1b ORF1a*-pET21/BL21 was generated by transformation of TTSuV1b ORF1a-pET21 plasmid into *E. coli* BL21. IPTG-induced bacteria lysate obtained from SDS-PAGE showed an overexpressed recombinant protein with a molecular weight of 22 kDa (Fig. 2a). Western blot analysis with an anti-Flag monoclonal antibody showed a specific band as expected (Fig. 2b), indicating successful expression of rTTSuV1b ORF1a. The target protein was recovered by gel extraction and showed a high purity in further SDS-PAGE examination (Fig. 2c).

### Indirect ELISA for detection of TTSuV1b

Optimal concentration of recombinant protein for plate coating and dilution factor for serum or HRP-labeled secondary antibody were determined using Checkerboard titrations. It was found that the OD values of TTSuV1b antibody positive serum samples were greater than 0.9 while the OD values of negative samples were smaller than 0.2. The same method was applied to detect the positive serum for CSFV, PCV2, Porcine parvovirus (PPV), PRRSV, and PRVgB antigens, and there was no positive reaction existed, implicating a good specificity of this method (Table 1). Moreover, a mean coefficient of variation (CV) between 0.9 and 1.93 % (Table 2) was observed in the experiments using the kits from same batch, while a mean CV between 4.18 and 6.91 % (Table 3) was seen in experiments done with kits from different batches, indicating excellent reproducibility.

**Table 1:** Specificity of ELISA method (OD value)

Coated antigen	CSFV, PCV2, PPV, PRRSV, or PRVgB Ab Positive serum					TTSuV1b Ab
	CSFV	PCV2	PPV	PRRS	PRV-gB	Positive serum
rTTSuV1b ORF1a	0.080	0.066	0.122	0.078	0.131	0.966

*Ab* antibody, *CSFV* Classical Swine Fever Virus, *PCV2* Porcine Circovirus type 2, *PPV* Porcine parvovirus, *PRRSV* Porcine Reproductive and Respiratory Syndrome Virus, *PRVgB* Pseudorabies Virus glycoprotein B, *TTSuV1b* Torque teno sus virus 1b, *ELISA* enzyme-linked immunosorbent assay, *OD* optical density

**Table 2:** Repeated experiments using indirect ELISA kits of same batch (OD value)

Serum samples	1	2	3	4	5	STDEV	MEAN	CV(%)
TTSuV1b Ab Positive serum	0.975	0.963	0.982	0.971	0.985	0.008	0.975	0.90 %
TTSuV1b Ab Negative serum	0.157	0.154	0.159	0.162	0.160	0.003	0.158	1.93 %

*STDEV* standard deviation, *CV* coefficient of variation

**Table 3:** Repeated experiments using indirect ELISA kits of different batch (OD value)

Antigens from different batch	Positive serum Inter-batch variation				Negative serum Inter-batch variation			
	1	2	3	4	1	2	3	4
1	1.172	0.983	1.025	0.989	0.158	0.131	0.137	0.130
2	1.090	0.969	1.046	1.071	0.171	0.152	0.134	0.132
3	1.105	1.040	1.159	1.081	0.166	0.131	0.115	0.116
4	1.038	1.022	1.042	1.091	0.165	0.157	0.119	0.122
5	1.072	1.005	1.095	1.004	0.147	0.142	0.125	0.116
MEAN	1.095	1.004	1.073	1.035	0.161	0.143	0.126	0.123
STDEV	0.049	0.028	0.054	0.044	0.009	0.011	0.009	0.007
CV (%)	4.50 %	2.86 %	5.08 %	4.27 %	5.77 %	8.31 %	7.49 %	6.15 %
Mean value of CV	4.18 %				6.91 %			

**Fig. 3:**
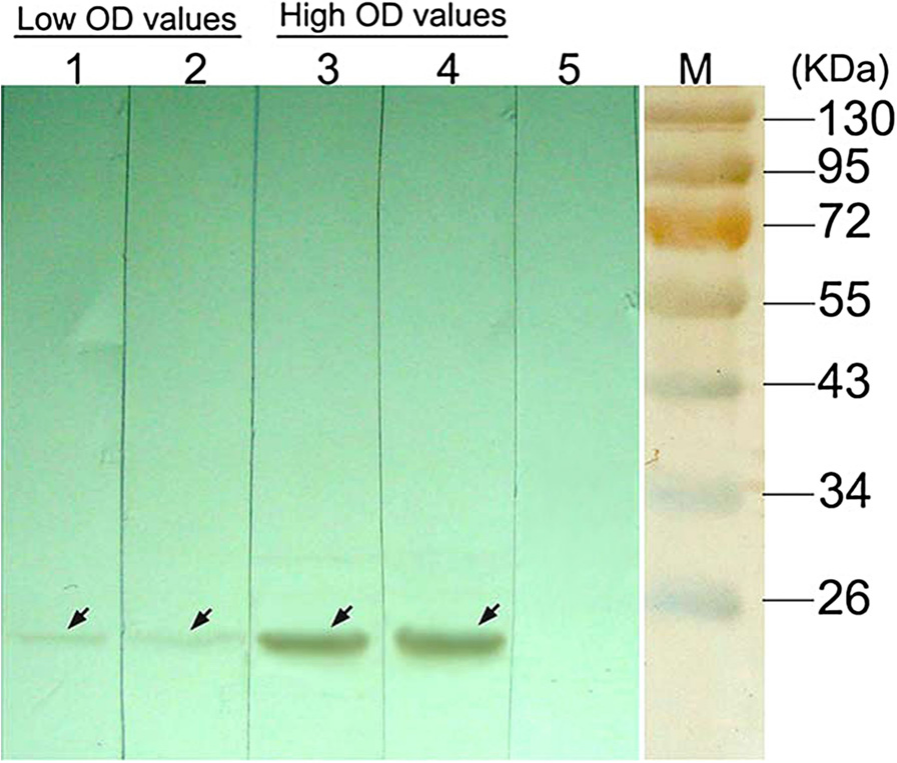
Western blot analysis of antibody against TTSuV1b in a typical serum sample. Lane 1: OD_450_ = 0.238; Lane 2: OD_450_ = 0.216; Lane 3: OD_450_ = 0.913; Lane 4:OD_450_ = 0.928. The targets are all marked with arrows

Serum samples with different levels of anti-TTSuV1b antibody identified by ELISA detection were further examined by Western blot analysis with rTTSuV1b ORF1a as antigen. Samples with high OD value (OD_450_ = 0.913, 1.118) showed strong signals, while those with low OD value (OD_450_ = 0.433, 0.416) showed weak signals (Fig. 3). Antibodies in serum specifically recognized a 22 kDa protein, which was of the same size as rTTSuV1b ORF1a. Western blot analysis further verified the specificity of ELISA method in detection of anti-TTSuV1b antibody. The truncated polypeptide TTSuV1b ORF1a was located at position 168–346 of TTSuV1b ORF1. These results indicated that specific antibody against this fragment existed in porcine serum.

**Fig. 4:**
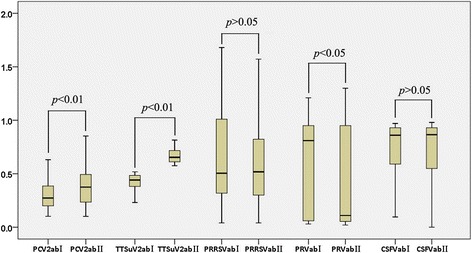
Box plots showing the Comparison of antibody levels in serum samples with low (Group I) or high (Group II) TTSuV1b virus

### Correlation between antibodies against different viruses in the serum

Antibodies against TTSuV1b, PRVgI, CSFV, PRRSV, or PCV2 in 212 serum samples were examined, and the correlation between the level of anti-TTSuV1b antibody and anti-PRVgI, anti-CSFV, anti-PRRSV, or anti-PCV2 antibodies in 212 serum samples were analyzed. The Pearson correlation coefficient r was as follows: r_TTSuV1b-PRV gI_ = −0.163, p = 0.015; r_TTSuV1b-CSFV_ = −0.136, p = 0.041; r_TTSuV1b-PRRSV_ = −0.065, p = 0.332; r_TTSuV1b-PCV2_ = 0.422, p = 0.007. According to Pearson correlation criteria, when 0.3 ≤ │r│ ≤ 0.5, the two groups were poorly correlated and when │r│ ≤ 0.3, there was a weak or no correlation. Accordingly, the serum antibody against TTSuV1b showed a certain low-extent correlation with anti-PCV2 antibody, while very weak or no correlation was observed between anti-TTSuV1b antibody and other examined antibodies.

### Correlation between TTSuV1b infection and four other antibodies against other viruses

The nucleic acids of TTSuV1b and PCV2 in 212 porcine serum samples were analyzed by PCR. The positive rate of TTSuV1b varied greatly among samples from different regions, ranging from 0 to 85.71 % (Table 4); while the infection of PCV2 was general, with a positive rate ranging from 20.69 to 71.43 % (Table 4). Serum samples were categorized into two groups according to the value of positive rate for TTSuV1b: Group I, low TTSuV1b; Group II, high TTSuV1b. In Group I, positive rate for TTSuV1b and PCV2 was 23.53 and 28.43 %, respectively; in Group II, the rate was 71.82 and 46.36 %, respectively. Statistical analysis indicated that positive rate for PCV2 in the two groups showed significant differences (p < 0.01, two tailed *t*-test), which increased significantly in samples with high TTSuV1b positive rate.

**Table 4:** Nucleic acid analysis of TTSuV1b and PCV2 in porcine serum samples

Group	Source of porcine sera	Positive rate	
		TTSuV1b	PCV2
Group I (low TTSuV1b level)	Zhaoqing	0.00 % (0/18)	27.78 % (5/18)
	Foshan	31.03 % (9/29)	20.69 % (6/29)
	Sihui	30.77 % (4/13)	30.77 % (4/13)
	Huizhou	31.58 % (6/19)	36.84 % (7/19)
	Heshan	21.7 % (5/23)	30.4 % (7/23)
	Total positive rate	23.53 % (24/102)	28.43 % (29/102)
Group II (high TTSuV1b level)	Xinhui	67.39 % (31/46)	54.35 % (25/46)
	Guangzhou	85.71 % (18/21)	28.57 % (6/21)
	Heyuan	57.14 % (4/7)	71.43 % (5/7)
	Yangjiang	72.22 % (26/36)	41.67 % (15/36)
	Total positive rate	71.82 % (79/110)	46.36 % (51/110)

The level of antibodies against PRVgI, CSFV, PRRSV, and PCV2 in serum from Group I and Group II were analyzed using *t*-test (Fig. 4). The levels of antibody against TTSuV1b and PCV2 showed significant difference between Group I and Group II (p < 0.01). The levels of TTSuV1b and PCV2 antibodies in Group I were significantly lower than those in Group II, indicating that after TTSuV1b and PCV2 infection, the viral load in serum was positively correlated with antibody levels. The comparison of level of antibody against PRVgI between Group I and Group II showed a statistically significant difference (p < 0.05); serum samples with low level of TTSuV1b antibody were accompanied by a low level of antibody against PRVgI (since antibody against PRVgI was detected by competitive ELISA, high OD value corresponded to low antibody level). As all serum samples were obtained from pigs that were not immunized with PRV vaccine, low level of PRVgI antibody represented low level of infection by wild PRV. Antibodies against PRRSV or CSFV in two groups were comparable (p > 0.05), with no statistical significance, implicating that in conventional pigs, the antibody level for PRRSV and CSFV were not significantly influenced by TTSuV1b infection.

## Discussion

In the present study, an ELISA method for detection of the TTSuV1b antibody using a truncated TTSuV1b capsid protein as antigen was established. The results indicated that the ELISA method was well reproducible with CV values falling in the normal range for intra-batch and inter-batch experiments; this method also showed good specificity, since the coated antigen did not cross-react with positive serum from other commercial kits. Further, Western blot analysis of serum antibodies confirmed that the ELISA method was reliable to use in the detection of TTSuV1b antibodies in porcine serum. In this study, the overexpressed truncated polypeptide TTSuV1b ORF1a was located at position 168–346 of TTSuV1b ORF1; immunological analysis indicated that this area contained the key antigenic epitope for B cells, which might induce the production of specific antibodies against the virus by the immune system. Antibody detection is an important approach to understand the immunological reaction between host and pathogens, and the level of antibody is directly correlated with the level of infection by pathogens under a certain condition. Huang et al. found that, when the load of TTSuV1b DNA in serum reached 104–107 copies/mL, the respective antibody titer was high, while when the load was low or there was no infection, the antibody titer was low [7].

TTSuVs are commensal with many other pathogens but do not directly lead to pathological changes [8]. Moreover, it has been previously reported that T cells might be the potential target cells infected by TTSuVs (Taiwan, 2014). However, the recent studies about potential pathogenicity of TTSuVs are based on another disease model [5, 9, 10, 19–23], in which the correlation between TTSuVs and a specific disease is determined by the load of TTSuVs in the serum or in the organs. Currently, TTSuVs has been thought to be related to PCV2 associated diseases (PCVAD). It has been reported that in PMWS caused by PCV2, virus load of TTSuV1b in porcine serum from pigs with the disease was significantly higher than that from pigs without the disease [5]. For piglets with PMWS, during the first 15 weeks, TTSuV1b viremia worsened over time [22]; the synergistic effect of TTSuV1a and PCV2 infection might exacerbate PMWS [21]. However, Lee et al. reported that there were no differences in the viral load of TTSuVs between porcine serum immunized with commercial PCV2 vaccine and serum of porcine not immunized with PCV2 vaccine [9]; later, Huang et al. implicated that TTSuV1a infection was not associated with PCVAD [24]. More biomarkers and further studies are needed to reveal the relationship between TTSuVs infection and PCVAD. In other diseases, Aramouni et al. found that virus load of TTSuV1b increased significantly after CSFV challenge [19]; TTSuV1a infection might impair or suppress the immune effect of PRRSV attenuated vaccine [23]. TTSuVs might impair the host immune function through a certain systematic mechanism.

In the present study, several antibodies of various viruses in 212 samples of porcine serum were examined and it was found that a certain correlation existed between TTSuV1b antibody and PCV2 antibody (r = 0.422); in high TTSuV1b group, PCV2 antibody and TTSuV1b antibody were both higher than those in low TTSuV1b group. Since these samples were obtained from pigs that were not immunized by PCV2 vaccine, the level of PCV2 antibody might reflect a wild PCV2 infection; with the nucleic acid analysis of the two viruses by PCR [5, 22], it was further confirmed that high TTSuV1b viremia was accompanied by high level of PCV2 infection. Notably, data from analysis of PRVgI antibody indicated that the level of PRVgI antibody in high TTSuV1b viremia group was significantly lower than that in low TTSuV1b viremia group, implicating high infection rate of PRV in high TTSuV1b viremia group, while the mechanism of interaction between the two viruses needs to be further validated. Detection of antibodies against PRRSV or CFSV indicated that, under natural conditions, the level of TTSuV1b viremia showed no obvious influence on the production of antibodies against PRRSV or CFSV, implicating that TTSuV1b infection might not affect infection or vaccine inoculation of PRRSV or CFSV.

## Conclusion

In the present study, the ELISA method was used to examine the level of anti-TTSuV1b antibody in 212 samples of porcine serum. In addition, the levels of antibodies against CSFV, PRV, PRRSV, or PCV2 were examined using commercial kits and analyzed the potential correlation between anti-TTSuV1b antibody and antibodies against four common viral infectious diseases. Examination of anti-TTSuV1b antibody in porcine serum with ELISA would serve as a supplementary approach for etiology analysis. Through statistical analysis of antibodies in serum, it was found that, under a natural feeding condition, infection of TTSuV1b showed a certain correlation and consistency with infection of PCV2, with a significantly higher level of PRV infection in pigs with high TTSuV1b viremia.

## Methods

### Construction of the expression plasmid

Bioinformatics tools were used to identify the ORF1 gene of a TTSuV1b strain [GenBank: JQ782385.1], including the encoded protein. According to the antigenicity analysis of TTSuV1b ORF1 protein, a truncated TTSuV1b ORF1, named TTSuV1b ORF1a, was cloned. A FLAG tag was added to the C-terminal of TTSuV1b ORF1a amino acid. The Primer sequences were as follows: TTV2 ORF1a-*Nde*IF: CGCCATATGAAAAAACAACACAAAATAGTA; TTV2 ORF1a-*Hind*IIIR: CCCAAGCTTtcaCTTATCATCGTCGTCCTTGTAGTCTCTTGATTTTGGATTTGTAGC. The PCR reaction was performed according to the manufacturer’s instructions (KOD-Plus, Toyobo): 94 °C, 5 min; 94 °C, 30s; 51 °C, 0 s; 68 °C, 30s for 30 cycles; 68 °C, 8 min. Later, the correct PCR product was recovered using agarose gel electrophoresis, followed by double restriction enzyme digestion, ligation with PET21b plasmid, and transformation into *Escherichia coli* (*E coli*) DH5a. Positive colonies were picked up by colony PCR method and the constructed TTSuV1b ORF1a-PET21b plasmid was validated by sequencing.

### Analysis of recombinant TTSuV1b ORF1a protein

The recombinant plasmid TTSuV1b ORF1a-PET21 was transformed into *E. coli* BL21, and cultured at 37 °C for 8 h, followed by colony PCR identification. The positive clones were seeded into 3 ml Liquid Amies medium and induced with 1 mM Isopropyl β-D-1-thiogalactopyranoside for 8 h. The transformed *E. coli* were collected by centrifugation, and then mixed with protein loading buffer, boiled for 15 min, and subjected to sodium dodecyl sulfate polyacrylamide gel electrophoresis (SDS-PAGE) for detection of the TTSuV1b ORF1a recombinant protein. Western blotting was used to detect the expression of rTTSuV1b ORF1a with a monoclonal anti-Flag antibody as the primary antibody. Proteins were recovered using Protein PAGE Recovery Kit (Sangon), and then dialyzed with Tris-buffered saline (TBS). The concentration of the purified TTSuV1b ORF1a recombinant protein was measured using bicinchoninic acid protein assay Kit (Pierce Biotechnology Inc.). The purified recombinant protein was stored at −80 °C.

### Indirect ELISA detection of serum antibody against TTSuV1b

Optimal antigen concentration, the dilutions of the sera and the horseradish peroxidase (HRP) conjugates were determined using checkerboard titrations. Negative serum and positive serum were determined according to the difference between the two optical density (OD) values. The indirect ELISA method was optimized as follows: Polystyrene plate (Nunc plates, Thermo Scientific) was coated with purified rTTSuV1b ORF1a, which had been diluted with coating buffer to a concentration of 200 ng/ml. After incubation at 37 °C for 2 h, each well was washed with 300 μl TBS and Tween 20 (TBST) for three times and blocked with 300 μl blocking reagent (Pierce Biotechnology Inc.) at 37 °C for 1 h. Subsequently, each well was filled with 100 μl serum (diluted with blocking reagent at the ratio of 1:40) to be tested. After incubation at 37 °C for 1.5 h, each well was washed with 300 μl TBST for three times. Then, 100 μl HRP-labeled goat anti-pig Immunoglobulin G (IgG, KPL) (diluted at the ratio of 1:2000) was added to each well and incubated at room temperature for 45 min. After being washed with 300 μl TBST for three times, 100 μl ELISA developing reagent was added and incubated at room temperature for 10 min. Reaction was ended with the addition of 100 μL 2 mol/l sulfuric acid in each well. Absorbance was measured at 450 nm. Experiment was considered to be effective when the average OD value of positive serum (Pm) and average OD value of negative serum (Nm) showed a difference beyond 0.30 (≥0.30). Positive or negative value was judged based on the cut-off value (COV) using the formula given below.

COV = Nm × 2.1 (when Nm was lower than 0.08, Nm was assigned to be 0.08). When the average OD of a sample was beyond COV (≥COV), the sample was judged to be positive (+); Otherwise, the sample was negative.

### Western blot assay of TTSuV1b–specific antibody in sera

The specificity of the serum antibody against TTSuV1b detected in ELISA method was verified using Western blot analysis as follows: purified rTTSuV1b ORF1a was subjected to SDS-PAGE, transformed to nitrocellulose (NC) membrane (Pall), and blocked with 10 % non-fat milk powder at 37 °C for 1 h. Then, porcine serum with high or low OD in ELISA detection were diluted at the ratio of 1:100, and incubated respectively with the NC membrane at 4 °C overnight. After being washed with TBST for three times, the NC membranes were incubated with HRP-labeled goat anti-pig IgG secondary antibody (KPL) with a dilution ratio of 1:2000 at 37 °C for 1 h. The NC membranes were then washed again with TBST for three times and subjected to color development with 3, 3′-diaminobenzidine reagent (Life technologies).

### PCV2 and TTSuV1b viremia analysis

The pigs that were not immunized with commercial vaccine for PCV2 or PRV were used in this analysis. A total of 212 blood samples were collected from the farms in Sihui, Conghua, Zhaoqing, Huizhou, and Maoming cities in Guangdong province, China. The sera were isolated, and the nucleic acid in the sera was extracted. Nucleic acid for PCV2 and TTSuV1b was detected using PCR. Samples were categorized into two groups according to the viremia of TTSuV1b: Group I, low TTSuV1b seropositivity; Group II, high TTSuV1b seropositivity.

### Analysis of the antibodies against TTSuv1b, PCV1, PRVgI, CSFB, or PRRSV in serum samples using ELISA method

The antibodies against PRV glycoprotein l (PRVgI), CSFV, PRRSV (IDEXX), or PCV2 (Ingenasa) in 212 porcine serum samples were detected according to the respective manufacturer’s instructions. The TTSuV1b-specific antibody in the sera was detected using ELISA method described above. Analysis on each sample was repeated three times. Statistical analyses were performed using the SPSS software as follows: 1. correlation analysis between the level of antibody against TTSuV1b and the levels of antibodies against PRVgI, CSFV, PRRSV, or PCV2; 2. difference of the antibodies against PRVgI, CSFV, PRRSV, or PCV2 and between Group I (low TTSuV1b seropositivity) and Group II (high TTSuV1b seropositivity).
